# Cereblon contributes to the development of pulmonary fibrosis via inactivation of adenosine monophosphate-activated protein kinase α1

**DOI:** 10.1038/s12276-021-00619-6

**Published:** 2021-05-17

**Authors:** Hyo Jae Kang, Kyung Jin Lee, Jisu Woo, Jiyeon Kim, Yun Kyu Kim, Chang-Hoon Lee, Chul-Gyu Yoo, Kyoung-Hee Lee

**Affiliations:** 1 Division of Pulmonary and Critical Care Medicine, Department of Internal Medicine, Uijeongbu Eulji Medical Center, Eulji University, Uijeongbu, Republic of Korea; 2 Department of Convergence Medicine, Asan Institute for Life Sciences, University of Ulsan College of Medicine, Asan Medical Center, Seoul, Republic of Korea; 3 Division of Pulmonary and Critical Care Medicine, Department of Internal Medicine, Seoul National University Hospital, Seoul, Republic of Korea; 4 Department of Internal Medicine, Seoul National University College of Medicine, Seoul, Republic of Korea

**Keywords:** Pathogenesis, Biochemistry

## Abstract

Pulmonary fibrosis is a progressive and lethal lung disease characterized by the proliferation and differentiation of lung fibroblasts and the accumulation of extracellular matrices. Since pulmonary fibrosis was reported to be associated with adenosine monophosphate-activated protein kinase (AMPK) activation, which is negatively regulated by cereblon (CRBN), we aimed to determine whether CRBN is involved in the development of pulmonary fibrosis. Therefore, we evaluated the role of CRBN in bleomycin (BLM)-induced pulmonary fibrosis in mice and in transforming growth factor-beta 1 (TGF-β1)-induced differentiation of human lung fibroblasts. BLM-induced fibrosis and the mRNA expression of collagen and fibronectin were increased in the lung tissues of wild-type (WT) mice; however, they were significantly suppressed in *Crbn* knockout (KO) mice. While the concentrations of TGF-β1/2 in bronchoalveolar lavage fluid were increased via BLM treatment, they were similar between BLM-treated WT and *Crbn* KO mice. Knockdown of CRBN suppressed TGF-β1-induced activation of small mothers against decapentaplegic 3 (SMAD3), and overexpression of CRBN increased it. TGF-β1-induced activation of SMAD3 increased α-smooth muscle actin (α-SMA) and collagen levels. CRBN was found to be colocalized with AMPKα1 in lung fibroblasts. CRBN overexpression inactivated AMPKα1. When cells were treated with metformin (an AMPK activator), the CRBN-induced activation of SMAD3 and upregulation of α-SMA and collagen expression were significantly suppressed, suggesting that increased TGF-β1-induced activation of SMAD3 via CRBN overexpression is associated with AMPKα1 inactivation. Taken together, these data suggest that CRBN is a profibrotic regulator and maybe a potential target for treating lung fibrosis.

## Lung disease: Putting the brakes on fibrosis

Interventions that target a regulatory protein called cereblon could help reduce the damage inflicted on the lungs by idiopathic pulmonary fibrosis (IPF). This incurable and generally fatal condition is associated with the accumulation of scar tissue in the lungs, which leads to the gradual loss of respiratory function. Researchers led by Kyoung-Hee Lee at Seoul National University Hospital in South Korea have now identified cereblon as a potentially important contributor to this scarring process. They found that cereblon regulates a complex metabolic pathway that ultimately contributes to production of fibrosis-related proteins in a mouse model of IPF. Genetically modified animals that lacked the gene encoding cereblon showed reduced accumulation of these proteins in their lungs. These results suggest that cereblon-inhibiting agents could potentially control the progression of IPF and help preserve lung function.

## Introduction

Idiopathic pulmonary fibrosis (IPF) is a chronic, progressive, and usually lethal lung disease characterized by fibrosis-induced destruction of the lung parenchyma. Several clinical trials on anti-inflammatory, antifibrotic, and anticoagulant agents as treatments for IPF have been performed. However, the results were mostly limited or showed no desirable effects^1^.

The pathogenesis of IPF involves epithelial and endothelial cell injuries of various causes. Injury mediates the secretion of various profibrotic mediators, such as fibroblast growth factors, metalloproteinases, and cytokines, and the leakage of blood factors into the airspace of the lungs. Subsequently, fibroblasts migrate into the injured area, and then myofibroblasts accumulate. As a result, in fibroblastic foci, myofibroblasts secrete inordinate amounts of extracellular matrix (ECM) proteins that destroy the lung parenchyma^1,2^.

Among the various profibrotic mediators, transforming growth factor-beta (TGF-β) is one of the key mediators involved in the pathogenesis of IPF. TGF-β levels are increased in the lung tissues of patients with IPF and in animal models of IPF. Moreover, the overexpression of active TGF-β leads to the development of persisting lung fibrosis, whereas blocking TGF-β signaling ameliorates lung fibrosis. TGF-β induces the migration, proliferation, and differentiation of mesenchymal cells and enhances the activation of fibroblasts and their differentiation into myofibroblasts. Free and active TGF-β binds to TGF-β receptor type I/II on the cell membrane. Type II receptors transphosphorylate type I receptors, which transduce the signal into cells by phosphorylating small mothers against decapentaplegic homolog 2 protein (SMAD2) and SMAD3. SMAD2/SMAD3 forms a complex with SMAD4 and translocate into the nucleus. In the nucleus, this complex interacts with the SMAD-binding element and then regulates the transcriptional response of several genes, including ECM protein-encoding genes^3,4^.

*CRBN*, which encodes cereblon, was first identified as a candidate gene associated with mild mental retardation in humans^5^. Therefore, the role of CRBN in the brain has been extensively studied^6–8^. CRBN is also expressed in several types of tissues, including lung, liver, kidney, heart, and brain tissues. Recent studies have explained the diverse roles of CRBN, including regulation of immunomodulatory imide drug-mediated E3 ligase activity in cancer, adenosine monophosphate-activated protein kinase (AMPK)-mediated energy metabolism regulation, and mental retardation-associated ion channel regulation^9^. CRBN binds to AMPKα1 and inhibits its activation^10^. AMPK is an important regulator of energy metabolism. AMPK dysfunction is involved in various metabolic diseases, such as diabetes, obesity, cardiac hypertrophy, and cancer^11^. AMPK is composed of one catalytic (α) subunit and two regulatory (β and γ) subunits. AMPKα is a protein that is known to interact with CRBN^10^. The binding of CRBN to AMPKα reduces the affinity between the α and γ subunits, which suppresses AMPK activity^10^. AMPK activation has been reported to reduce the development of pulmonary, cardiac, and renal fibrosis^12–14^. Since CRBN negatively regulates AMPK activity, CRBN may be a novel therapeutic target for lung fibrosis. However, whether CRBN affects lung fibrosis and its underlying mechanism of pathogenesis are not known. The objective of this study was to investigate the role of CRBN in bleomycin (BLM)-induced lung fibrosis in mice and TGF-β1-induced differentiation of lung fibroblasts.

## Materials and methods

### Mice and BLM-induced pulmonary fibrosis

Female wild-type (WT) C57BL/6 mice were purchased from Koatech Laboratory Animal Company (Pyeongtaek, Korea), and *Crbn* knockout (KO) mice on the C57BL/6 genetic background were kindly donated by Dr. Kyung Jin Lee (Asan Medical Center, Seoul, Korea). All mice used were of the same sex, were 6–8 weeks of age (20–22 g), and were housed in the animal facility of Seoul National University Hospital, Seoul, South Korea, under specific pathogen-free barrier conditions. The animal experiments were approved by the Institutional Animal Care and Use Committee (number 17-0133-S1A0(2)) of the Seoul National University Hospital. The mice were anesthetized and administered 5 units/kg BLM (Nippon Kayaku, Tokyo, Japan) in 100 μl of saline or saline alone via intratracheal injection.

### Lung histology

The right lungs of the mice were fixed in 4% paraformaldehyde solution (Santa Cruz Biotechnology Inc., Santa Cruz, CA, USA), embedded in paraffin, and sectioned at a thickness of 4 µm. The slides were subjected to Masson’s trichrome staining to examine the extent of peribronchial collagen deposition.

### Multiplex bead assay for measuring TGF-β1/2 levels in bronchoalveolar lavage fluid (BALF)

On day 11 after BLM administration, the lungs of terminally anesthetized mice were lavaged using 1 ml of cold phosphate-buffered saline. The BALF was centrifuged at 1500 rpm at 4 °C for 10 min, and the supernatants were collected to measure TGF-β1/2 levels. TGF-β1/2 levels in BALF were determined using a commercially available Bio-Plex Pro^TM^ Cytokine Assay Kit (Bio-Rad, Hercules, CA, USA) according to the manufacturer’s instructions.

### Isolation of RNA and amplification via quantitative real-time polymerase chain reaction

Total RNA was isolated using an RNeasy kit (Qiagen, Hilden, Germany). cDNA was synthesized using the Reverse Transcription System (Promega, Madison, WI, USA). Power SYBR Green PCR Master Mix (Applied Biosystems, Carlsbad, CA, USA) was used for amplification. The primers used in the study were as follows: mouse *Crbn* (forward (fwd): 5′-AGC ATG GTG CGG AAC TTA-3′; reverse (rev): 5′-ATC TCT GCT GTT GTC CCA-3′), mouse collagen (fwd: 5′-TGC CGT GAC CTC AAG ATG TGC C-3′; rev: 5′-CAT CCA CAA GCG TGC TGT AGG TG-3′), mouse collagen type 1 (fwd: 5′-CCT CCT GAC GCA TGG CCA AGA-3′; rev: 5′-TGC ACG TCA TCG CAC ACA GCC-3′), mouse fibronectin (fwd: 5′-ACC GTG TCA GGC TTC CGG GT-3′; rev: 5′-ACG GAA GTG GCC GTG CTT GG-3′), mouse glyceraldehyde-3-phosphate dehydrogenase (*Gapdh)* (fwd: 5′-TCC CTC AAG ATT GTC AGC AAT G-3′; rev: 5′-AGA TCC ACA ACG GAT ACA TTG G-3′), human *CRBN* (fwd: 5′-CAG TCT GCC GAC ATC ACA TAC-3′; rev: 5′-GCA CCA TAC TGA CTT CTT GAG GG-3′), and human *GAPDH* (fwd 5′-GAA GGT GAA GGT CGG AGT C-3′; rev 5′-GAA GAT GGT GAT GGG ATT TC-3′).

### Cells and reagents

Human lung IMR-90 fibroblasts (Korean Cell Line Bank, Korea) were maintained in Dulbecco’s modified Eagle’s medium (ThermoFisher Scientific, Waltham, MA, USA) containing 10% fetal bovine serum and 1% penicillin/streptomycin at 37 °C under 5% CO_2_ and 95% air. Recombinant human TGF-β1 proteins (240-B) were purchased from R&D Systems (Minneapolis, MN, USA). Antibodies used for protein detection included anti-phospho-SMAD3 (p-SMAD3) (Ser423/425), anti-p-AMP-activated protein kinase α1 (p-AMPKα1) (Thr172) (Cell Signaling Technology, Danvers, MA, USA), anti-CRBN (Merck, Darmstadt, Germany), anti-α-smooth muscle actin (α-SMA) (Abcam, Cambridge, MA, USA), anti-SMAD, anti-GAPDH (Santa Cruz Biotechnology Inc., Santa Cruz, CA, USA), and anti-collagen I (SouthernBiotech, Birmingham, AL, USA) antibodies. Alexa Fluor 488- and Alexa Fluor 555-conjugated antibodies and Hoechst 33342 were purchased from ThermoFisher Scientific. Metformin was obtained from Sigma-Aldrich (St. Louis, MO, USA).

### Transfection of plasmid vectors

Transfection with a control (pcDNA3.1) or CRBN overexpression vector (pcDNA3.1-hCRBN) was performed using Lipofectamine® 2000 transfection reagent (ThermoFisher Scientific) according to the manufacturer’s instructions.

### Western blotting

Total cellular extracts were prepared using 1× Cell Lysis Buffer (Cell Signaling Technology) supplemented with 1 mM phenylmethylsulfonyl fluoride. The protein concentration was determined using the Bradford assay (Bio-Rad). The protein from the cellular extracts was separated via sodium dodecyl sulfate-polyacrylamide gel electrophoresis and transferred onto Hybond ECL nitrocellulose membranes (ThermoFisher Scientific) for 100 min at 90 V. The membranes were blocked with 5% skim milk in 1× Tris-buffered saline containing 0.1% Tween 20 for 1 h at room temperature. After successive washes, the membranes were incubated with horseradish peroxidase-conjugated secondary antibodies for 1 h. The blots were developed using the West Pico Western Blot Detection Kit (ThermoFisher Scientific).

### Immunofluorescence staining

IMR-90 cells grown in 35 mm dishes were fixed in ice-cold methanol and incubated with an anti-AMPKα1 or anti-CRBN antibody (diluted 1:100 in 3% bovine serum albumin (BSA)) for 24 h. The cells were subsequently incubated with an Alexa Fluor 488- or Alexa Fluor 555-conjugated antibody (diluted 1:100 in 3% BSA) for 30 min. After successive washes, the cells were analyzed using a fluorescence microscope (EVOS FL microscope, ThermoFisher Scientific).

### Statistical analysis

Statistical analysis was performed using GraphPad software version 5 (GraphPad Software, San Diego, CA, USA). The data were analyzed using a two-tailed unpaired t-test or the Mann–Whitney *U* test as appropriate to determine significance. Data from the in vitro cell experiments are presented as the mean ± standard deviation. Data from the in vivo mouse experiments are expressed as the mean ± standard error. A *p*-value of <0.05 was considered significant.

## Results

### CRBN is essential for the development of BLM-induced lung fibrosis in mice

**Fig. 1:**
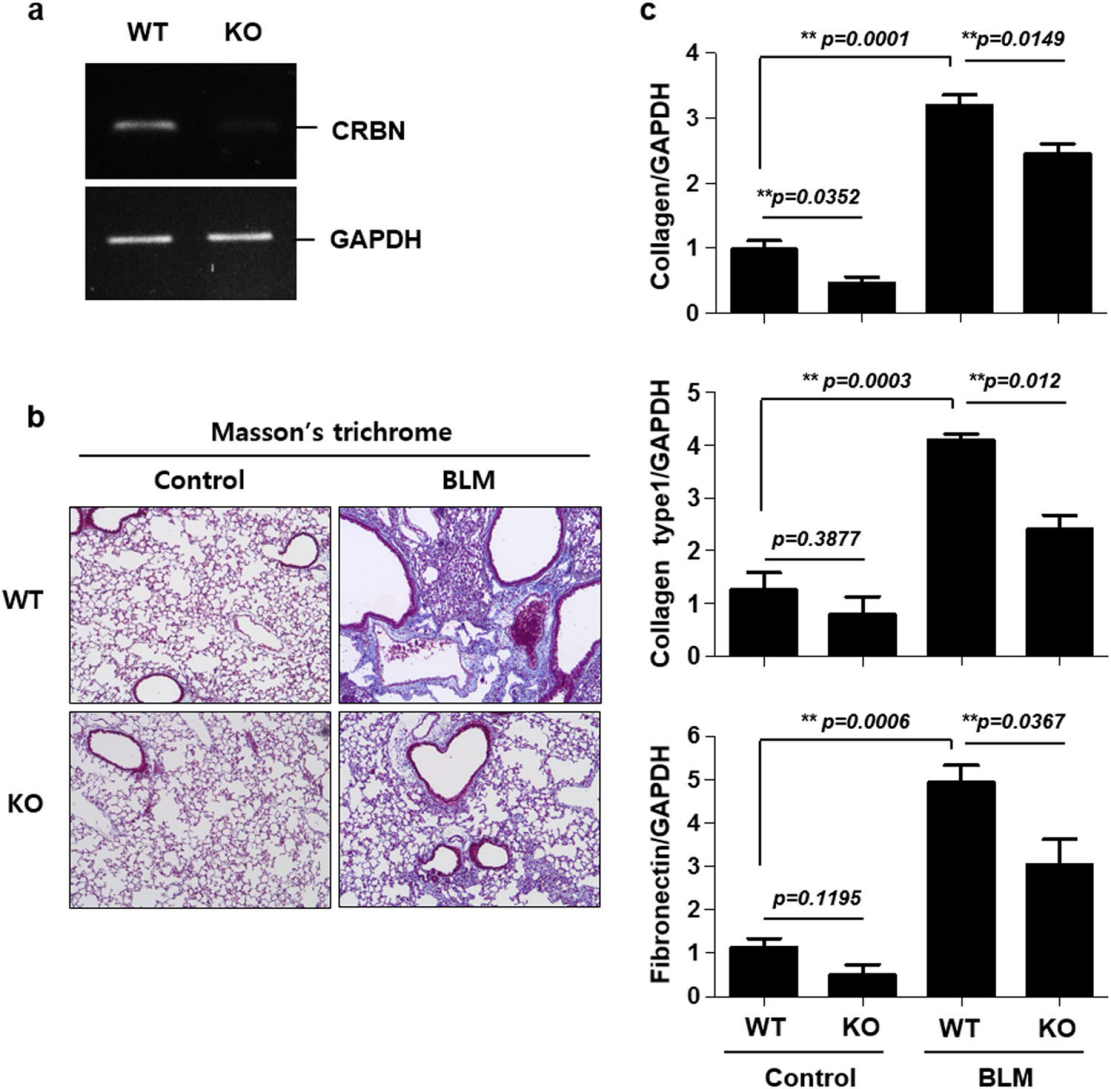
CRBN is necessary for the development of BLM-induced lung fibrosis in mice. **a** RT-PCR analysis of *Crbn* and *Gapdh* expression in lung tissues from WT and *Crbn* KO C57BL/6 mice. **b**, **c** WT and *Crbn* KO mice were treated with intratracheal instillation of BLM (5 units/kg) or saline (control) on day 0. Representative photographs of lungs from WT and *Crbn* KO mice on day 11 after intratracheal instillation of BLM or saline. **b** Sections were stained using Masson’s trichrome staining. **c** Real-time PCR analysis of total collagen, collagen type 1, fibronectin, and GAPDH expression in the lung tissues of WT and *Crbn* KO mice (control *n* = 3; BLM-treated mice, *n* = 4). The data are presented as the mean ± standard error. CRBN cereblon, BLM bleomycin, *Crbn* KO mice, cereblon knockout mice, WT mice wild-type mice.

We first evaluated the role of CRBN in the development of fibrosis in mice via BLM treatment. *Crbn* KO was confirmed via RT-PCR analysis (Fig. 1a). Masson’s trichrome staining showed that collagen deposition in the lungs was increased after BLM treatment. Notably, collagen deposition was lower in the lungs of BLM-treated *Crbn* KO mice than in those of BLM-treated WT mice (Fig. 1b). In accordance with the histological data, the expression levels of fibrotic markers such as total collagen, collagen type 1, and fibronectin, which were measured via real-time PCR, were lower in BLM-treated *Crbn* KO mice than in BLM-treated WT mice (Fig. 1c). These results indicated that CRBN might be at least partly essential for the development of BLM-induced lung fibrosis in mice.

### CRBN is not associated with the BLM-induced increase in TGF-β levels

**Fig. 2:**
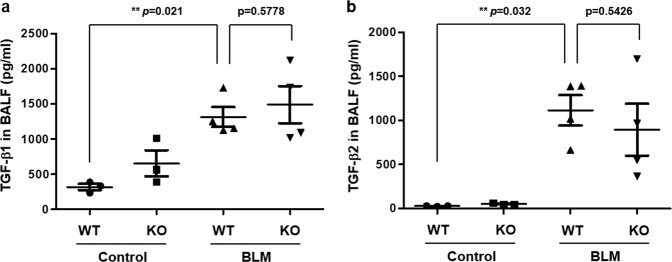
CRBN is not associated with the BLM-induced increase in TGF-β levels. **a**, **b** The TGF-β1/2 level in BALF was determined via multiplex bead assays (control *n* = 3; BLM-treated mice, *n* = 4). The data are presented as the mean ± standard error. CRBN cereblon, BLM bleomycin, TGF**-**β transforming growth factor beta, BALF bronchoalveolar lavage fluid.

Since TGF-β1, especially macrophage-derived TGF-β1, is essential for the development of pulmonary fibrosis^15^, we measured the concentration of TGF-β1/2 in BALF via multiplex bead assays. While the concentration of TGF-β1/2 in the BALF of BLM-treated WT and *Crbn* KO mice was significantly higher than that in the BALF of saline-treated WT and *Crbn* KO mice (Fig. 2a, b), there was no difference in the TGF-β1/2 concentration in BALF between BLM-treated WT and *Crbn* KO mice (Fig. 2a, b). These findings indicated that CRBN might not be associated with the BLM-induced increase in TGF-β levels.

### CRBN positively regulates the SMAD pathway, resulting in the differentiation of fibroblasts into myofibroblasts

**Fig. 3:**
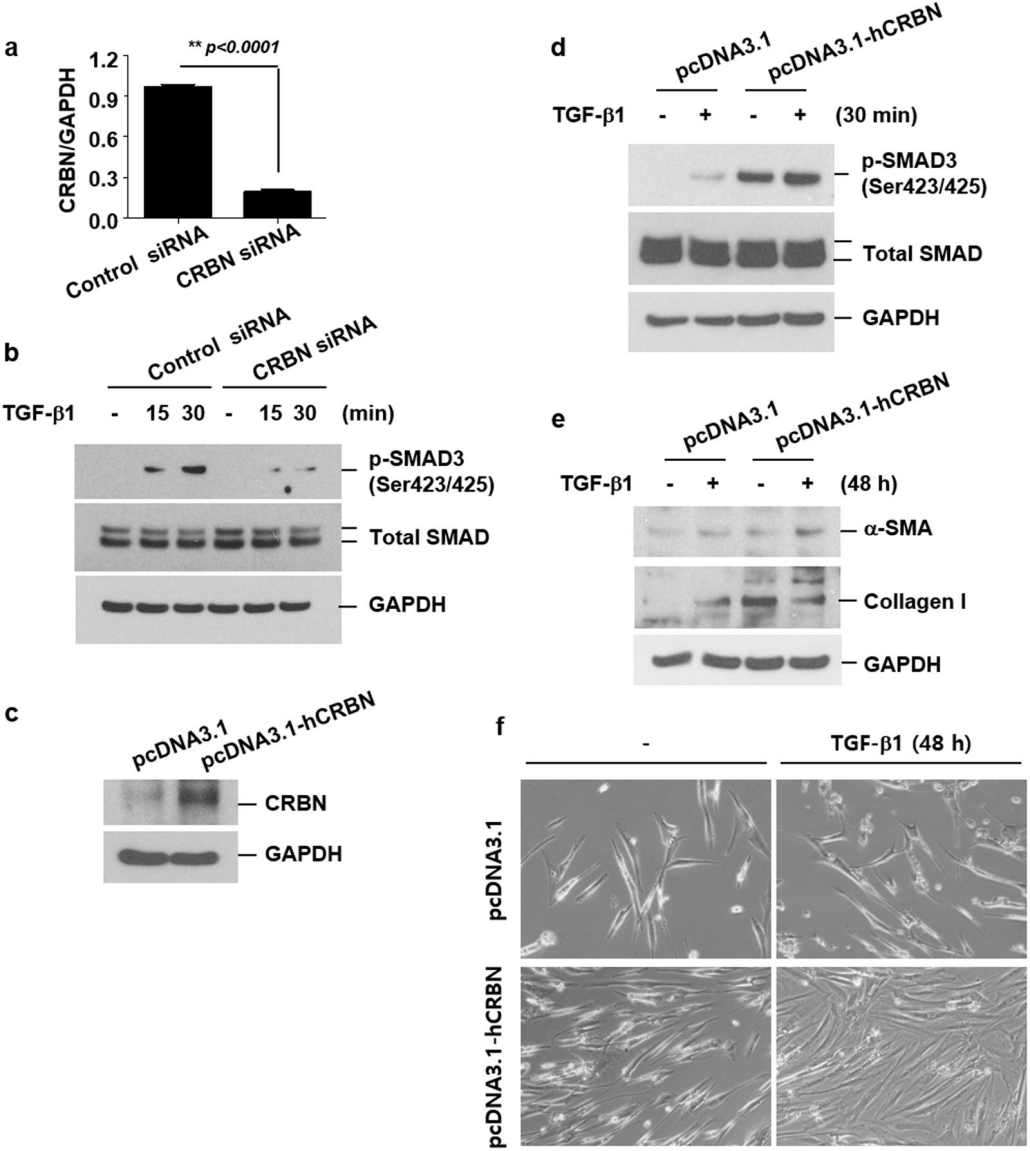
CRBN positively regulates the SMAD pathway, resulting in the differentiation of fibroblasts into myofibroblasts. **a**, **b** IMR-90 cells were transiently transfected with control or CRBN siRNAs. Forty-eight hours after transfection, the cells were treated with TGF-β1 (10 ng/ml) for the indicated times. **a** Real-time PCR analysis of CRBN and GAPDH expression. **b** Total cellular extracts were subjected to western blot analysis to measure the levels of phosphorylated SMAD3 (p-SMAD3) (Ser423/425), total SMAD, and GAPDH. **c**–**f** IMR-90 cells were transiently transfected with control or CRBN overexpression vectors. Forty-eight hours after transfection, the cells were treated with TGF-β1 (10 ng/ml) for the indicated times. **c**–**e** Total cellular extracts were subjected to western blot analysis to measure the levels of CRBN, p-SMAD3 (Ser423/425), total SMAD, α-SMA, collagen I, and GAPDH. f Representative photographs of control and CRBN-overexpressing IMR-90 cells 48 h after TGF-β1 treatment. The results are representative of three independent experiments. CRBN cereblon, SMAD small mothers against decapentaplegic, siRNA small-interfering RNA, TGF**-**β1 transforming growth factor-beta 1.

Since TGF-β1 is known to be associated with the differentiation of fibroblasts into myofibroblasts by activating the SMAD pathway^3,4^, we examined the role of CRBN in the activation of the SMAD pathway in human lung fibroblasts (IMR-90 cells). TGF-β1 increased the level of the active phosphorylated form of SMAD3 (Ser423/425, p-SMAD3) in control small-interfering RNA (siRNA)- and control plasmid-transfected cells (Fig. 3b, d). As shown in Fig. 3a, CRBN knockdown suppressed the TGF-β1-induced increase in p-SMAD3 levels (Fig. 3b). In contrast, as shown in Fig. 3c, CRBN overexpression not only increased basal p-SMAD3 levels but also enhanced the TGF-β1-induced increase in p-SMAD3 levels (Fig. 3d). These results indicated that CRBN might have positively regulated the SMAD pathway in lung fibroblasts. We next examined the role of CRBN in the differentiation of fibroblasts into myofibroblasts via the action of TGF-β1 in human lung fibroblasts. Overexpression of CRBN enhanced the TGF-β1-induced upregulation of α-SMA and collagen expression (Fig. 3e). Furthermore, CRBN overexpression increased the TGF-β1-induced proliferation of IMR-90 cells (Fig. 3f). These results indicated that CRBN might positively regulate the SMAD pathway, thereby resulting in the differentiation of fibroblasts into myofibroblasts.

### The profibrotic effect of CRBN might be due to the inactivation of AMPKα1

**Fig. 4:**
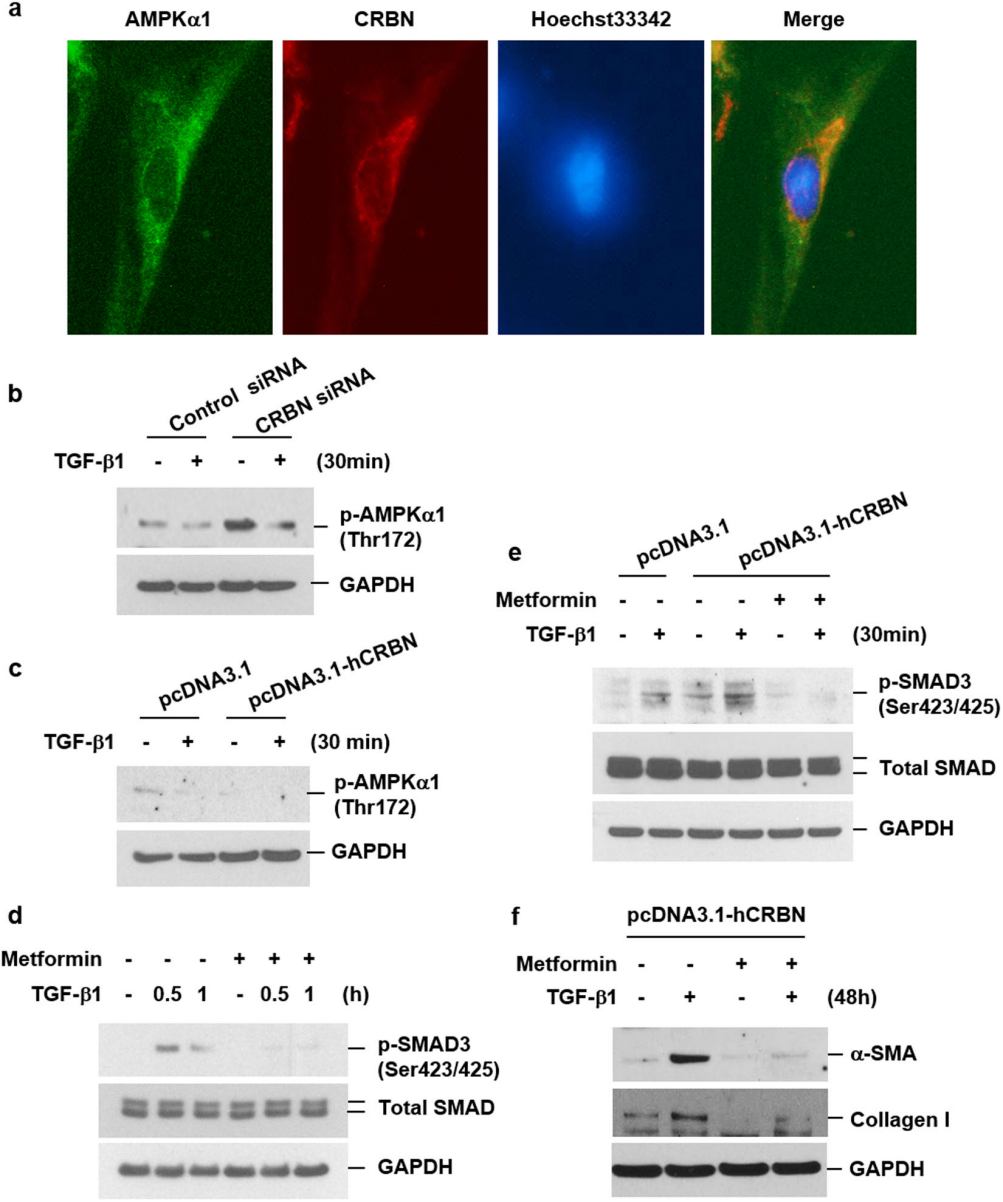
The profibrotic effects of CRBN might be due to the inactivation of AMPKα1. **a** IMR-90 cells were double-stained with specific antibodies against CRBN and AMPKα1, and the expression patterns were visualized using an EVOS FL microscope. AMPKα1, CRBN, Hoechst 33342, and merged signals are shown as green, red, blue, and yellow, respectively; magnification, ×200. **b** Cells were transiently transfected with control siRNAs or CRBN siRNAs. **c** Cells were transiently transfected with control or CRBN overexpression vectors. Forty-eight hours after transfection, the cells were treated with TGF-β1 (10 ng/ml) for 30 min. Total cellular extracts were subjected to western blotting for p-AMPKα1 and GAPDH. **d** IMR-90 cells were pretreated with metformin (5 mM) for 1 h and then stimulated with TGF-β1 (10 ng/ml) in the presence or absence of metformin for the indicated times. **e**, **f** IMR-90 cells were transiently transfected with control or CRBN overexpression vectors. Forty-eight hours after transfection, the cells were pretreated with metformin (5 mM) for 1 h and then incubated with TGF-β1 (10 ng/ml) in the presence or absence of metformin for the indicated times. Total cellular extracts were subjected to western blotting for p-SMAD3 (Ser423/425), total SMAD, α-SMA, collagen I, and GAPDH. The results are representative of three independent experiments. AMPKα1 adenosine monophosphate-activated protein kinase alpha 1, p-SMAD3 phosphorylated small mothers against decapentaplegic protein 3.

Since AMPK activation has been reported to suppress BLM-induced lung inflammation and fibrosis^12^, we investigated whether the profibrotic effects of CRBN described above are related to AMPK. Therefore, we first evaluated whether CRBN was colocalized with AMPK. Immunofluorescence staining of IMR-90 lung fibroblasts showed that endogenous AMPKα1 and CRBN were colocalized in the cytoplasm (Fig. 4a). We next examined whether CRBN inactivates AMPKα1. CRBN knockdown increased basal active phosphorylated AMPKα1 (Thr172, p-AMPKα1) levels, and CRBN overexpression decreased basal p-AMPKα1 levels (Fig. 4b, c). In contrast, regardless of whether CRBN was overexpressed or knocked down, TGF-β1 decreased the levels of p-AMPKα1 (Fig. 4b, c). Therefore, CRBN might have inactivated AMPKα1 but did not mediate the TGF-β1-induced inactivation of AMPKα1. To determine whether CRBN mediates lung fibrosis by inactivating AMPKα1, we tested the effect of metformin (a pharmaceutical activator of AMPK) on TGF-β1-induced activation of the SMAD pathway and collagen deposition in CRBN-overexpressing cells. Metformin treatment inhibited the TGF-β1-induced increase in p-SMAD3, α-SMA, and collagen levels in normal fibroblasts (Fig. 4d–f), and this effect was not influenced by CRBN overexpression. These results indicated that increased CRBN is essential for TGF-β1-induced activation of the SMAD pathway, which is mediated via AMPKα1 inactivation. Overall, these findings suggested that the profibrotic effect of CRBN might be due to the inactivation of AMPKα1.

## Discussion

**Fig. 5:**
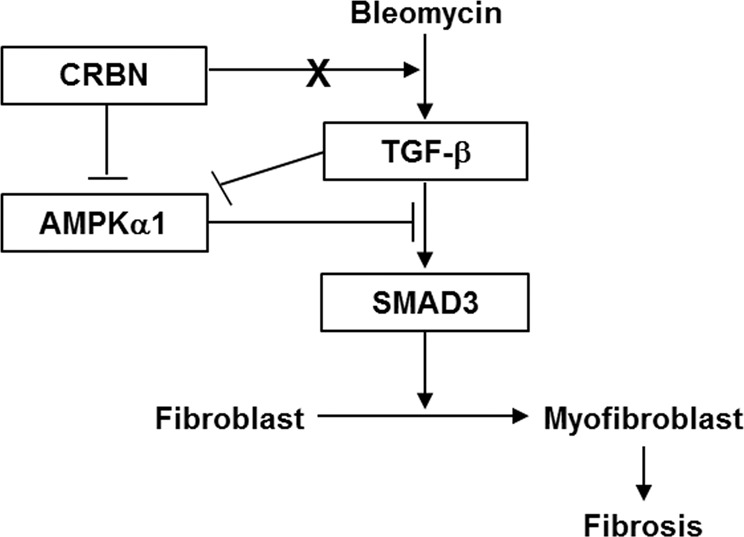
Schematic diagram of the role of CRBN in lung fibrosis. BLM treatment induces lung fibrosis by mediating the release of TGF-β and activation of the SMAD pathway. CRBN does not increase the BLM-induced release of TGF-β. However, CRBN increases the TGF-β-induced activation of SMAD3 by inhibiting AMPKα1 phosphorylation in lung fibroblasts. Increased activation of SMAD3 by CRBN results in the differentiation of fibroblasts into myofibroblasts.

In this study, we showed that CRBN deficiency may suppress the development of BLM-induced pulmonary fibrosis in mice in vivo and inhibit the TGF-β1-induced activation of lung fibroblasts in vitro by suppressing SMAD3 phosphorylation. Moreover, we found that the increased activation of SMAD3 by CRBN is mediated via AMPKα1 inactivation (Fig. 5).

BLM-induced lung fibrosis was suppressed in *Crbn* KO mice. The expression levels of ECM protein-encoding genes were significantly lower in the lung tissues of BLM-treated KO mice than in those of BLM-treated control mice. TGF-β1 is known to increase collagen synthesis and deposition via fibroblasts; macrophage-derived TGF-β1 is required for pulmonary fibrosis^3,15^. Therefore, TGF-β1 is a central therapeutic target for different types of fibrosis. However, the expression levels of TGF-β1 in BALF were not suppressed in BLM-treated *Crbn* KO mice, which suggested that CRBN exert a profibrotic effect on the next step after TGF-β1 release. Therefore, we investigated the role of CRBN in TGF-β1-treated lung fibroblasts.

Fibrosis is characterized by the accumulation of fibroblasts that release excessive amounts of ECM. The TGF-β1/SMAD signaling pathway directly regulates the expression of genes encoding ECM proteins such as collagen, fibronectin, and α-SMA. As expected, the knockdown of CRBN suppressed the activation of the TGF-β1/SMAD pathway by inhibiting SMAD3 phosphorylation. In contrast, overexpression of CRBN enhanced TGF-β1-induced SMAD3 activation, subsequently increased the expression levels of ECM proteins, and promoted fibroblast growth. Regarding the association between CRBN and the SMAD pathway, CRBN is known as a substrate receptor that forms an E3 ubiquitin ligase complex with cullin-4A and damaged DNA-binding protein 1^16^. This complex ubiquitinates several proteins, resulting in protein degradation. Neither CRBN knockdown nor overexpression affected the expression levels of total SMAD protein. The decrease in p-SMAD3 expression in CRBN siRNA-transfected cells seemed not to be due to the role of CRBN as a substrate receptor for the SMAD protein.

AMPK is well known to be a metabolic regulator, and reduction in its activity is an important risk factor for the development of organ fibrosis^17–19^. Mice receiving the AMPK activator metformin show significantly reduced lung fibrosis^12,20^. Since CRBN inactivates AMPKα1, we determined whether the inhibitory effect of CRBN knockdown on the SMAD pathway is mediated via AMPK activation. While CRBN knockdown increased the basal level of p-AMPKα1, TGF-β1-mediated downregulation of p-AMPKα1 expression was not completely blocked via CRBN knockdown. Although CRBN inactivated AMPKα1, the TGF-β1-induced inactivation of AMPKα1 did not appear to be mediated by CRBN. When CRBN-overexpressing fibroblasts were treated with metformin, the increases in TGF-β1-induced activation of SMAD3 and expression of α-SMA and collagen via CRBN overexpression were completely suppressed. These data implied that CRBN does not directly regulate the activity of SMAD3 and that CRBN may contribute to pulmonary fibrosis via the inactivation of AMPKα1 in lung fibroblasts. Similarly, the AMPK activator 5-aminoimidazole-4-carboxyamide ribonucleoside (AICAR) was shown to reverse TGF-β1-induced SMAD signaling in vascular smooth muscle cells^21^ and suppress SMAD3-dependent transcription and myofibroblast transdifferentiation^22^. However, the mechanisms of action of AMPK activators appear to be cell-type specific. In kidney fibroblasts, although AICAR suppresses myofibroblast activation, it does not block TGF-β1-induced activation of SMAD2/3^23^.

In this study, *Crbn* KO only partially reduced the deposition of ECM proteins in the lungs of mice treated with BLM. This might have been because other intracellular signaling cascades, including the non-SMAD (SMAD-independent) TGF-β signaling pathway, are involved in the upregulation of ECM protein expression^24^ and because CRBN might not block all pathways associated with the expression of ECM genes. Several types of lung cells, such as epithelial cells, macrophages, endothelial cells, and fibroblasts, express CRBN^25–27^. In evaluating the role of CRBN in lung fibrosis, we tested the effect of cigarette smoke extract (CSE) on CRBN expression in lung epithelial cells since epithelial cell injury is considered as an initiating event in lung fibrosis and cigarette smoking is the most consistent environmental risk factor. Neither the treatment of lung epithelial cells with CSE nor intratracheal instillation of CSE (once a week for 8 weeks) altered the expression levels of CRBN (data not shown). In addition, we assessed the relationship between CRBN and the CSE-induced increase in the expression of aging markers. Knockdown of CRBN did not affect the CSE-induced increase in the expression of an aging marker (p21) in lung epithelial cells (data not shown). Further detailed studies elucidating the relationship between CRBN and known risk factors for lung fibrosis are needed.

In conclusion, we demonstrated that disruption of the CRBN gene prevented the development of BLM-induced lung fibrosis by suppressing SMAD3 phosphorylation in lung fibroblasts. This study provides evidence for the therapeutic potential of targeting CRBN and indicates that CRBN antagonists (inhibitors) may be useful for the treatment of lung fibrosis.
